# Effect of Hematite Doping with Aliovalent Impurities on the Electrochemical Performance of α-Fe_2_O_3_@rGO-Based Anodes in Sodium-Ion Batteries

**DOI:** 10.3390/nano10081588

**Published:** 2020-08-12

**Authors:** Vincenza Modafferi, Claudia Triolo, Michele Fiore, Alessandra Palella, Lorenzo Spadaro, Nicolò Pianta, Riccardo Ruffo, Salvatore Patanè, Saveria Santangelo, Maria Grazia Musolino

**Affiliations:** 1 Dipartimento di Ingegneria Civile, dell’Energia, dell’Ambiente e dei Materiali (DICEAM), Università Mediterranea di Reggio Calabria, 89122 Reggio Calabria, Italy; vincenza.modafferi@unirc.it (V.M.); claudia.triolo@unirc.it (C.T.); 2 Dipartimento di Scienza dei Materiali, Università di Milano Bicocca, 20125 Milano, Italy; m.fiore11@campus.unimib.it (M.F.); n.pianta@campus.unimib.it (N.P.); riccardo.ruffo@unimib.it (R.R.); 3 Istituto di Tecnologie Avanzate per l’Energia (ITAE) del Consiglio Nazionale delle Ricerche (CNR), 98126 Messina, Italy; alessandra.palella@itae.cnr.it (A.P.); lorenzo.spadaro@itae.cnr.it (L.S.); 4 Dipartimento di Scienze Matematiche e Informatiche, Scienze Fisiche e Scienze della Terra (MIFT), Università di Messina, 98166 Messina, Italy; patanes@unime.it; 5 Consorzio Interuniversitario Nazionale per la Scienza e Tecnologia dei Materiali (INSTM), 50121 Firenze, Italy

**Keywords:** sodium ion batteries, hematite, doping, reduced graphene oxide, nanocomposite

## Abstract

The effect of the type of dopant (titanium and manganese) and of the reduced graphene oxide content (rGO, 30 or 50 wt %) of the α-Fe_2_O_3_@rGO nanocomposites on their microstructural properties and electrochemical performance was investigated. Nanostructured composites were synthesized by a simple one-step solvothermal method and evaluated as anode materials for sodium ion batteries. The doping does not influence the crystalline phase and morphology of the iron oxide nanoparticles, but remarkably increases stability and Coulombic efficiency with respect to the anode based on the composite α-Fe_2_O_3_@rGO. For fixed rGO content, Ti-doping improves the rate capability at lower rates, whereas Mn-doping enhances the electrode stability at higher rates, retaining a specific capacity of 56 mAhg^−1^ at a rate of 2C. Nanocomposites with higher rGO content exhibit better electrochemical performance.

## 1. Introduction

Today, with the increasing energy demand and the exhaustion of conventional fossil fuels reserves, humankind is facing a global energy challenge and serious environmental issues, such as air pollution and global warming. This has sparked an intensive research on the development of a sustainable, environmentally friendly and renewable energy resource, like solar, wind, wave and hydrogen energy [1,2]. However, the intermittent nature and the often unpredictable availability of these energy resources restrict their utility as a direct and reliable power sources. To integrate renewable energy sources into an electric grid, the development of inexpensive and high-efficiency energy storage/conversion devices has become one of the major challenges [3]. In this context, energy storage based on rechargeable batteries represents one of the most promising technologies due to its high round-trip efficiency, low maintenance, flexible power and long cycle life, as well as pollution free [4,5,6]. Among various electrochemical energy storages (EESs) available in the market, lithium-ion batteries (LIBs) have emerged as the main contender, since their commercialization by Sony Corp. in the 1991. They play a dominant role in the sector of portable electronic devices (e.g., laptop, cellular phone, digital cameras and MP3 players) and are considered as the best option to power next generation electric vehicles (EVs), hybrid electric vehicles (HEVs) and plug-in hybrid electric vehicles (PHEVs) [7,8,9,10,11]. The consequent increasing demand for LIBs, especially in the automotive sector, gives rise about the limited availability of global lithium resources coupled with a geographically uneven distribution, which could ultimately raise the lithium price and hinder large-scale applications of LIBs [12]. The sodium-ion batteries (SIBs) potentially seem to be a more sustainable alternative to LIBs in the field of grid-scale energy storage, due to greater abundance and wide geographical distribution of sodium in the Earth’s crust, its lower cost and similar chemistry to lithium [13,14,15,16,17].

Nonetheless, the commercialization of SIBs is hampered by several technological barriers still to overcome, such as lower energy/power densities and a shorter cycle life than LIBs. The larger radius (0.102 nm) and molar mass (22.99 g/mol) of sodium ion as compared to those of the lithium ion (0.076 nm and 6.94 g/mol, respectively) negatively affect the Na^+^ ions diffusion kinetics into the host materials [18,19]. As a consequence, finding and developing suitable host frameworks for SIBs with larger channels and interstitial sites to accommodate Na^+^ ions, allowing reversible and rapid ions insertion/extraction, are still a challenge.

For what concerns cathode materials, encouraging results in relation to capacity, cycling stability and charge retention have been achieved, as extensively described in the literature [20,21]. A large number of materials, mainly including Na_x_MO_2_ layer transition metal oxides, (0 < x ≤ 1; M = Cr, Co, Mn, Ni, Fe, V, Cu and their mixtures) [13,20,22,23], polyanionic compounds (such as phosphates, pyrophosphates, sulphates, silicates and sodium super-ionic conductors) [24,25,26,27,28,29,30]. Prussian blue-based frameworks [31,32], transition metal fluorides [33] and organic [34] has been investigated as promising cathodes. Conversely, the identification of a suitable anode material still remains a critical issue for the successful development of SIBs. Graphite, the traditional anode material for commercial LIBs, is not suitable for SIBs since it does not intercalate sodium ions to appreciable extent, delivering a low reversible capacity (35 mAhg^−1^). Theoretical calculations show that the interlayer distance of graphite (0.34 nm) is too small and lower than the critical distance requested for Na^+^ ions insertion (0.37 nm) [35,36]. Many studies have been focused on other non-graphitic carbonaceous materials, like as hard carbon, carbon nanofibers, carbon nanowires and hollow carbon nanospheres [37,38,39,40,41]. Hard carbon materials, characterized by a large interlayer distance and disordered structure, which favor Na^+^ cation insertion–extraction, have been reported to have reversible capacities of 225–300 mAhg^−1^ [42,43,44]. However, non-graphitic carbonaceous materials as anodes suffer from the high reversibility and capacity loss and poor cycling stability, making their still far from practical application. Sodium alloy with elements of the 14th (such as Na_15_Sn_4_, 847 mAhg^−1^ [45,46] and Na_15_Ge_4_, 369 mAhg^−1^ [47]) and 15th (such as Na_3_Sb, 660 mAhg^−1^ [48]) group have been also proposed as a possible alternative because of their high theoretical capacity and appropriate potential of Na^+^ insertion. Phosphorus forms Na_3_P by reacting electrochemically with sodium, achieving a theoretical specific capacity of 2596 mAhg^−1^ at a relatively safe working potential (0.4 V vs. Na^+^/Na), which significantly exceeds that of any other SIBs anode material reported in the literature [49,50]. However, the practical application of alloy-based materials as well as P in SIBs is hampered by drastic volume expansion (>300%) occurring during cycling that will lead to the mechanical disintegration of the electrode materials and a fast capacity fading.

Recently, many metal oxides (MOs) such as copper oxide [51,52], nickel oxide [53,54], tin oxide [55,56], manganese oxides [57,58], cobalt oxides [59,60,61] and iron oxides [62,63,64] are gathering attention as alternative anode materials for SIBs, due to their high reversible capacities and low cost. Among MOs, which store sodium ions via a conversion mechanism, iron (III) oxide (Fe_2_O_3_) is particularly appealing owing to its chemical stability, high theoretical specific capacity (1007 mAhg^−1^), easy synthesis, non-toxicity and environmental friendliness [55,64,65]. Unfortunately, Fe_2_O_3_ suffers from the drawbacks of most of the MOs, such as a limited cycle life and low Coulombic efficiency attributed to a drastic volume variation, although much lower if compared to alloying materials, and unavoidable particles aggregation occurring during the sodiation/desodiation process. An effective method to tackle these problems is to embed Fe_2_O_3_ nanoparticles (NPs) into conductive carbon framework (e.g., carbon nanotubes, graphene, graphene oxide and others) for fabricating nanostructured hybrids. These hybrids not only significantly improve the electrical conductivity of anodes but also effectively buffer the large volume change during the charge/discharge process [66,67,68,69]. Graphene sheets, generally obtained by a chemical reduction of graphene oxide (GO), are used successfully as a carbon matrix for nanocomposites, because of their excellent electrical conductivity, high specific surface area, rapid heterogeneous electron transfer and superior mechanical flexibility [70,71]. The synergistic effect between reduced graphene oxide (rGO) and loaded Fe_2_O_3_ NPs effectively induced superior performance. For example, iron oxide nanocrystals anchored onto graphene nanosheets, synthesized by a nanocasting technique and used as an anode in SIBs, exhibited a specific capacity of about 400 mAhg^−1^ for more 200 cycles at 100 mAg^−1^ [72]. On a different approach Yang’s group prepared α-Fe_2_O_3_ nanorods supported on rGO composite by a simple seed-assisted hydrothermal growth route, obtaining a discharge capacity of 332 mAhg^−1^ at a current density of 0.2 C over 300 cycles [73]. Fe_2_O_3_ on rGO nanomaterial, fabricated via microwave-assisted hydrothermal method by Liu et al., delivered a reversible capacity of 289 mAhg^−1^ at a current density of 50 mAg^−1^ after 50 cycles [74].

In previous papers we have reported the good electrochemical performance as anode materials in SIBs of the Fe_2_O_3_@rGO nanocomposites, synthesized by one-step solvothermal route, a very simple and scalable method [75,76]. This technique allows one to anchor Fe_2_O_3_ on the graphene sheets in one single step and in the absence of any chemical reducing agent.

In this work, nanostructured composites, consisting of hematite nanoparticles doped with aliovalent elements, anchored on reduced graphene oxide (α-Fe_2_O_3_:D@rGO) were synthesized by a one-step solvothermal method and tested as anode material for SIBs. To our best knowledge, very few studies have so far been reported on the synthesis of doped Fe_2_O_3_@rGO for SIBs. Both tetravalent and bivalent dopants, such as Ti^4+^ and Mn^2+^ are evaluated with the main purpose to improve the electrochemical performance of the α-Fe_2_O_3_@rGO nanocomposites. The morphology, microstructure and composition of the synthesized nanostructures are also analyzed by a combination of complementary techniques to investigate how the presence of the dopant influences the nanostructure, the crystalline phase and the electrochemical activity of the resulting oxide anchored on graphene sheets.

## 2. Materials and Methods

### 2.1. Materials

Potassium permanganate (KMnO_4_, ACS reagent purity ≥99.0%), graphite powder (Purum, particle size ≤ 0.1 mm), iron (II) acetate (Fe(CH_3_COO)_2_, 95% purity), manganese (II) acetate tetrahydrate (Mn(CH_3_COO)_2_·4H_2_O, purity: ≥99%), *N*-Methyl-2-pyrrolidone (ACS reagent, 99% purity), sodium perchlorate (NaClO_4_, ACS Reagent, purity ≥98%) and fluoroethylene carbonate (99.9% purity) were purchased from Sigma-Aldrich (Darmstadt, Germany). Sodium nitrate (NaNO_3_, 99.5% purity), sulfuric acid (H_2_SO_4_, 95–97% purity) and propylene carbonate (anhydrous, purity ≥99.9%) were obtained from Merck (Darmstadt, Germany). Carlo Erba reagents (Milan, Italy) have supplied hydrogen peroxide (H_2_O_2_, 30 wt %) and ethanol 96°. Hydrochloric acid (HCl, 36 wt %) and titanium (IV) isopropoxide (Ti[OCH(CH_3_)_2_]_4_, purity: >97%) were purchased from Alfa Aesar (Kandel, Germany). All reactants and solvents were employed directly without further purification. Distilled water was used throughout the experiments.

### 2.2. Synthesis of Graphene Oxide and Nanocomposites

GO was obtained from graphite powder via a modified Hummers method [77]. In brief, graphite, sodium nitrate and concentrated H_2_SO_4_ were mixed and stirred in an ice bath. KMnO_4_ was slowly dropped into the mixture under stirring and cooling. The mixture was stirred for 2 h at 35 °C. The temperature was then increased to about 98 °C and distilled water was added. Subsequently, H_2_O_2_ was slowly added and the suspension color changed from brown to yellow. The as-obtained GO, recovered through centrifugation, was thoroughly washed with HCl aqueous solution and with distilled water, in sequence. Finally, the solid was dried at 50 °C in a vacuum oven for 2 days. Further details can be found elsewhere [76].

As reported in the Supplementary Materials (SM), the obtainment of GO and its reduction to rGO during the solvothermal process were verified by carrying out micro-Raman spectroscopy (MRS), X-ray powder diffraction (XRPD) and X-ray photoelectron spectroscopy (XPS) analyses. The corresponding changes in morphology were documented by scanning electron microscopy (SEM).

The as-prepared GO was utilized for the synthesis of hematite-based nanomaterials via the solvothermal method. The effect of:

(i) The hematite doping with aliovalent impurities (Ti or Mn) and of;
(ii) The nominal rGO content of the nanocomposites (50 or 30 wt %);

on their physicochemical properties and electrochemical performance as active SIB anode materials was investigated.

For this purpose, nanocomposites based on pure hematite (α-Fe_2_O_3_@rGO) were synthesized by dispersing 200 mg of GO in 60 mL of ethanol under sonication and by adding 10 mL of a solution of iron (II) acetate with a proper molarity, namely 0.20 M or 0.29 M for 50 and 30 wt % nominal rGO content, respectively. The reaction mixture was stirred at 80 °C for 10 h and, subsequently, was transferred in a Teflon beaker and sealed in a stainless steel autoclave for the solvothermal treatment at 170 °C for 3 h. After centrifugation, washing with ethanol and distilled water for several times, the resulting product was dried in air. Further details can be found elsewhere [76].

The same procedure was followed to prepare the nanocomposites based on Ti- and Mn-doped hematite (α-Fe_2_O_3_:Ti@rGO and α-Fe_2_O_3_:Mn@rGO, respectively). Titanium (IV) isopropoxide and manganese (II) acetate tetrahydrate were used as titanium and manganese sources, respectively. Regardless of the nominal hematite content of the nanocomposites (50 or 70 wt %), the dopant:iron atomic ratio was kept nominally constant at 1:20.

In the following, the nanocomposites are coded as α-Fe_2_O_3_(:D)@rGO-*w*, where D stands for the dopant (if any) and *w* denotes the nominal rGO content, expressed in weight percent. The real rGO content was estimated by carrying out a thermogravimetric analysis (TGA).

### 2.3. Nanocomposite Characterization

The produced nanocomposites were analyzed by means of a combination of techniques. TGA for the estimation of the rGO content was performed by using a NETZSCH STA 449C instrument (NETZSCH-Gerätebau GmbH, Selb, Germany). A few milligrams of each sample were heated in air from 25 to 1000 °C at a rate of 10 °C/min. The results obtained are reported in Table S1. The texture and morphology of the samples were investigated by SEM. A Phenom Pro-X scanning electron microscope (Thermo Fisher Scientific Co., Waltham, MA, USA) equipped with an energy-dispersive X-ray (EDX) spectrometer was utilized for this purpose.

The crystalline phase of the iron oxide was identified by means of MRS and XRPD analyses. MRS measurements were performed at room temperature (RT) and in air by means of a confocal microscope (NTEGRA—Spectra SPM from NT-MDT Spectrum Instruments, Moscow, Russia) coupled to a solid-state laser operating at 2.33 eV (532 nm). In order to avoid local heating of the samples, a variable ND filter was utilized to set the laser power at the sample surface at the value of 250 µW. A 100× objective was used both for the excitation and for the Raman scattering signal collection, which was dispersed by a 600 lines mm^−1^ grating and detected by a cooled CCD Camera (ANDOR iDus, Abingdon, UK). The XRPD patterns were recorded at RT by using the Ni β-filtered Cu-K_α_ radiation (λ = 0.15404 nm) at 40 KV. Analyses were registered in the 10–80° 2θ-angle range at a scan speed of 0.5 °/min. The JCPDS database of reference compounds was used to identify a diffraction peak, while the Rietveld refinements were useful to study doping-induced distortions in the hematite lattice.

The surface chemical composition of the samples and chemical environment of the component species were evaluated by X-ray photoelectron spectroscopy (XPS). Spectra were recorded using a Physical Electronics GMBH PHI 5800-01 spectrometer (Physical Electronics GmbH, Munich, Germany), equipped with a monochromatic Al-K_α_ source (1486.6 eV) with a power beam of 300 W. The pass energy for determination of the oxidation state and concentration of surface species was 11.0 eV and 58.0 eV, respectively. The binding energies were set taking the C 1*s* peak at 284.8 eV as reference. To identify and quantify the surface species the deconvolution of the high-resolution photoelectron spectra of C 1*s*, O 1*s*, Fe 2*p*, Ti 2*p* and Mn 2*p* core levels was performed. The areas measurement under the photoelectron peaks weighed by the relative sensitivity factors allowed us to determine the elemental concentrations. Table S2 reported the data obtained.

### 2.4. Electrochemical Measurement

Galvanostatic cycling with potential limitation (GCPL) were obtained by using the BioLogic VSP-300 multichannel potentiostat/galvanostat (BioLogic Sciences Instruments, Seyssinet-Pariset, France) on two-electrode coin cells CR2032 in an argon filled glove box (MBraun). Sodium metal foil was applied as both a counter and reference electrode. The working electrode was prepared according to the doctor blade technique. The active material (namely, nanocomposite α-Fe_2_O_3_@rGO, α-Fe_2_O_3_:Ti@rGO or α-Fe_2_O_3_:Mn@rGO), the carbon matrix (Super P, MM Carbon, Alfa Aesar, Kandel, Germany) and the polymer binder (polyacrylic acid *M*_w_ 450,000, Sigma Aldrich, Darmstadt, Germany) in the weight ratio 8:1:1, respectively, were mixed with *N*-Methyl-2-pyrrolidone solvent to form a slurry, which was spread on a copper foil and dried at 80 °C under vacuum overnight.

A pretreatment at 800 °C under argon atmosphere was performed on the conductive carbon matrix, to remove the adsorbed water and impurities, reducing the typical irreversibility that occurs during the first charge/discharge in such systems. The working electrode was then roll-pressed by using disks with a diameter of 16 mm. The active material loading was about 1 mg/cm^2^. A 1 M solution of NaClO_4_ in anhydrous propylene carbonate with 2 wt % fluoroethylene carbonate additive was employed as an electrolyte. The GCPL tests were carried out in the voltage range of 0.01 V and 3.00 V vs. Na/Na^+^ at different current rates.

Electrochemical impedance spectroscopy (EIS) measurements were performed in three electrodes Swagelok cells equipped with Na foils at both counter and reference electrodes. All the electrodes had a diameter of 1 cm. The spectra were obtained at OCV (2.6 V vs. Na^+^/Na) after a small charge input (cut-off 2.5 V) at C/20 to enable the electrochemical process without altering the materials. The sinusoidal voltage stimulus was 10 mV in the frequency range from 200 kHz to 100 mHz.

## 3. Results and Discussion

### 3.1. Nanocomposite Physicochemical Properties

**Figure 1:**
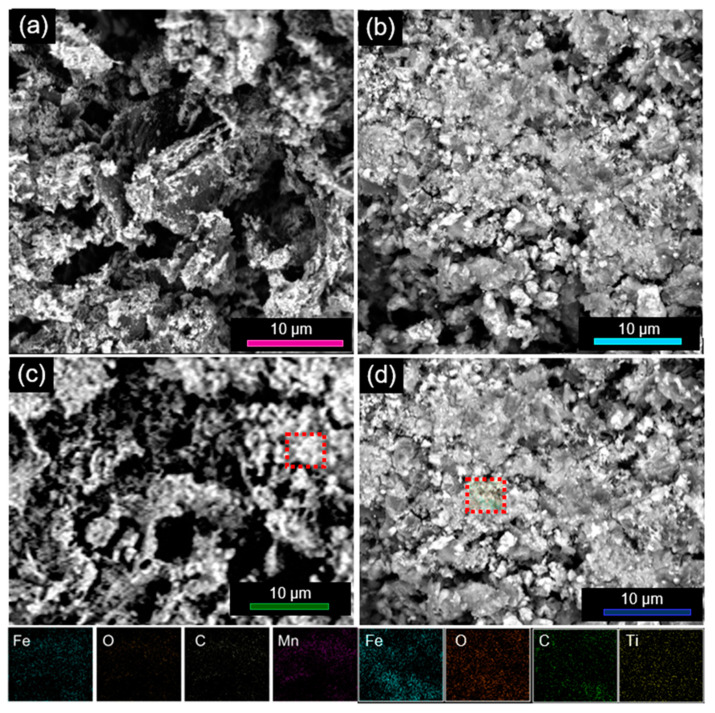
(**a**–**d**) Morphology and (**c**,**d**) elemental composition nanocomposites. The shown SEM images refer to samples (**a**) α-Fe_2_O_3_@rGO-50, (**b**) α-Fe_2_O_3_:Ti@rGO-50, (**c**) α-Fe_2_O_3_:Mn@rGO-30 and (**d**) α-Fe_2_O_3_:Ti@rGO-30; the elemental maps refer to the region marked by a red dashed rectangle of samples (**c**) α-Fe_2_O_3_:Mn@rGO-30 and (**d**) α-Fe_2_O_3_:Ti@rGO-30.

Figure S1 displays the morphologic changes that the solvothermal treatment produces on the GO support, which, as previously shown [76], undergoes thermal reduction to rGO without the need of any reducing agent (see below). The morphology of the nanocomposites is shown in Figure 1. All of them exhibit indented and rough texture, which is associated with the presence of thin rGO sheets. Regardless of the use of doping agents during the preparation, the iron oxide NPs appeared uniformly dispersed onto the rGO sheets (Figure 1a–c). The results of the EDX analysis (Figure 1c,d) proved that the dispersion of carbon, oxygen, iron and dopant (if any) within the samples was spatially uniform.

Table 1 reports the composition of the samples, as determined via TGA (Figure S3). For further details see Table S1 in the SM. Three main temperature ranges were singled out in the thermogravimetric profiles, corresponding to the release of physically absorbed water molecules (*T* ≤ 150 °C) [78,79] and to the complete combustion of the rGO flakes, starting from their edges (150 °C < *T* ≤ 400 °C) [80] and subsequently extending to the graphenic planes (400 °C < *T* ≤ 780 °C) [81,82]. For *T* > 780 °C, the residual mass does not change any more. It comes into view that the measured rGO content always exceeds the nominal one in the case of nanocomposites α-Fe_2_O_3_(:D)@rGO-30, whereas the opposite situation occurs in the case of α-Fe_2_O_3_(:D)@rGO-50.

**Figure 2:**
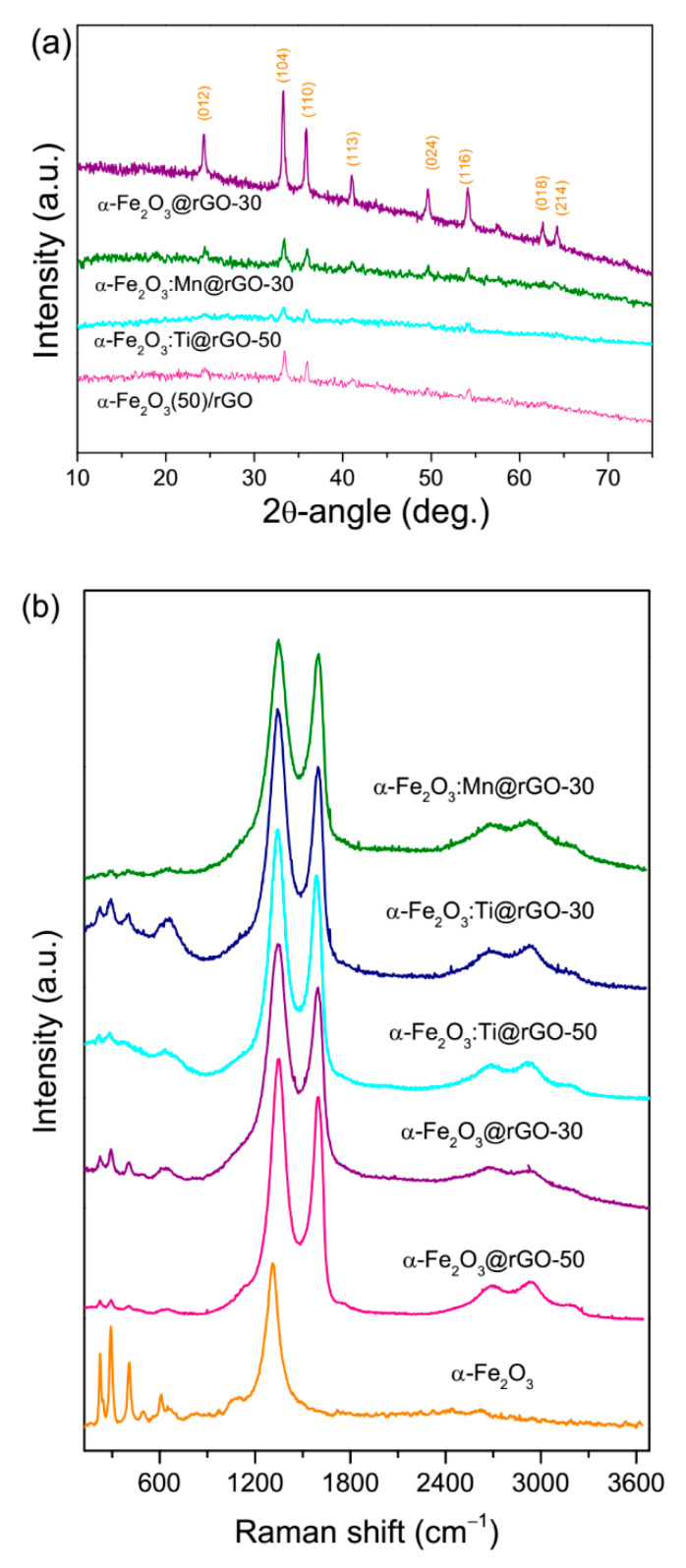
(**a**) XRPD patterns and (**b**) Micro-Raman spectra of the nanocomposites. The micro-Raman spectrum of the unsupported α-Fe_2_O_3_ nanoparticles, previously synthesized by the same method [75], is reported for comparison purposes.

Figure 2 and Figure S2a,b show the results of the XRPD and MRS analyses carried out on the considered nanomaterials, in order to ascertain the reduction of GO to rGO upon solvothermal treatment, to identify the crystalline phase of the iron oxide formed and to assess the graphitization degree of the carbon component in the nanocomposites.

Only the signals from the oxide were visible in the XRPD patterns of the nanocomposites (Figure 2a), while the Raman fingerprint of their carbon component dominated their micro-Raman spectra (Figure 2b). The D-band (at 1346 cm^−1^, for 2.33 eV excitation) arose from the *A*_1g_ breathing modes of the C atoms organized in hexagonal rings and was activated by finite size effects and by lattice defects breaking the translational symmetry of the graphitic layers; the G-band (at 1590 cm^−1^) originated from the *E*_2g_ in-plane stretching of all the pairs of C atoms [83]. Its frequency position was sensitive to the charge transfer, local distortions and hybridization changes of the C–C bonding, as well as to the oxidation degree of the π network, whereas the D/G intensity ratio (*I*_D_/*I*_G_), which intensifies with the increase of the structural disorder, commonly monitors the density of the C*sp*^2^ defects [83,84]. The changes observed by comparing the micro-Raman spectra of pristine and thermally treated GO.support (Figure S2a) indicate a diminishing of its oxidation degree and a partial restoration of the π network (Figure S2a), proving its reduction to rGO, in line with previous results [75,76] and expectations [82]. For further details see SM. The evolution of the XRPD pattern (Figure S2b) confirmed this finding. The narrow peak peculiar to GO was replaced by the very broad band typical of disordered graphitic carbons and *d*-spacing drastically reduced, approaching the theoretical value for graphene (0.34 nm) [82]. Further details are reported in the SM.

As for the iron oxide component, the XRPD analysis (Figure 2a) revealed that, regardless of the use of doping agents during the preparation, rhombohedral hematite (α-Fe_2_O_3_, JCPDS card No. 33-0664) was the only crystalline phase formed. No signals from other iron oxide phases, such as maghemite (γ-Fe_2_O_3_), magnetite (Fe_3_O_4_) or wüstite (FeO) were detected. The estimation of the average size of the α-Fe_2_O_3_ crystallites from the most intense diffraction peak via the Scherrer equation (Table 1) provides values varying in the ranges 27–28 nm and 17–29 nm for nanocomposites based on pure- and doped-hematite, respectively. This suggests that the nominal rGO content was not influential. Conversely, the presence of dopant may strongly affect the size of the hematite crystallites, in agreement with the literature [85,86]. In the present case, a drastic diminishing was observed only in the case of titanium (17–18 nm). Doping-induced distortions in the hematite lattice were evidenced by the Rietveld refinements on (104) and (110) diffraction peaks. In the composite α-Fe_2_O_3_@rGO-30, the lattice parameters of the unit cell of hematite were a = 5.01 Å and c = 13.71 Å. A decrease in the unit cell dimensions, in both in-plane and out-of-plane directions, was observed in the composite α-Fe_2_O_3_:Mn@rGO-30 (a = 4.99 Å and c = 13.69 Å), in agreement with the results reported by other authors [87,88]. This finding was understood as the effect of the smaller size of the Mn ions with an oxidation state higher than 3 [89] (see below) compared to Fe^3+^ (0.65 Å). Additionally, Ti substitution of Fe in the hematite lattice caused lattice parameter contraction (a=4.10 Å and c=13.70 Å). A possible reason for the slighter contraction with respect to that observed in the composite α- Fe_2_O_3_:Mn@rGO-30 is that when Ti substitutes Fe, it is reduced from Fe^3+^ to Fe^2+^ with an atomic radius increase (0.78 Å) [89,90].

The results of Raman scattering measurements (Figure 2b) confirmed the indications emerging from the XRPD analysis. The spectrum of unsupported iron oxide nanoparticles previously produced via the same method [75] is featured by six peaks at 224, 244, 293, 407, 496 and 609 cm^−1^, which respectively correspond to the Raman-allowed *A*_1g_(1), *E*_g_(1), unresolved *E*_g_(2)-*E*_g_(3), *E*_g_(4), *A*_1g_(2) and *E*_g_(5) phonon modes of crystalline α-Fe_2_O_3_ [91,92,93,94]. In addition, a very weak peak centered at 658 cm^−1^ and a very intense asymmetric peaking at 1315 cm^−1^ are observed, which respectively originate from the presence of heteroatoms, lattice defects and/or reduced grain size activating the IR-active *E*_u_ mode [91,92] and from two-magnon scattering on antiparallel close spin sites [93,94]. In the nanocomposites, the latter feature overlapped to the D-band arising from the rGO support, whereas only the most intense Raman-allowed phonon modes were clearly visible in the lowest frequency region of the spectra. Their intensity is generally higher in nanocomposites with smaller rGO content, as expected.

**Figure 3:**
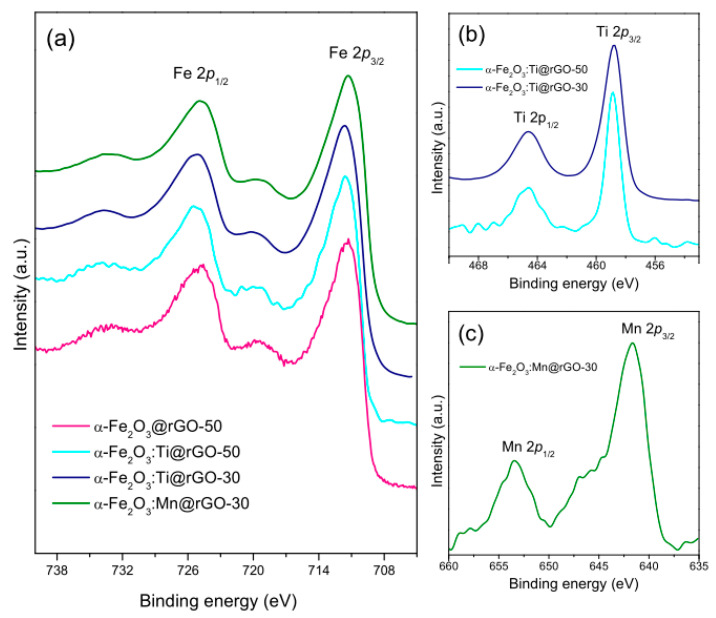
XPS spectra of (**a**) Fe^0^ 2*p*, (**b**) Ti^0^ 2*p* and (**c**) Mn^0^ 2*p* core levels in the nanocomposites.

Figure 3 and Figure S2c display the main results of the XPS analysis carried out to investigate the chemical environment of the component species at the nanomaterial surface and to ascertain the reduction of GO to rGO, respectively. The remarkable difference between the C 1*s* high resolution spectra of the pristine and the thermally treated GO support (Figure S2c) evidence the removal of oxygenated functionalities upon the solvothermal reaction, in line with the indications provided by XRPD and MRS analyses. As a result, the O_C_/C atomic ratio underwent a remarkable lowering (see SM). Further details can be found in the SM and ref. [75].

In all the nanocomposites, the high-resolution photoelectron spectra of the Fe 2*p* core level (Figure 3a) were featured by two prominent peaks at binding energies (BEs) of 724.8–725.5 eV and 711.3–711.6 eV (Table S2). They correspond to the 2*p*_1/2_ and 2*p*_3/2_ spin-orbit components of Fe^3+^ in α-Fe_2_O_3_ [95,96,97,98,99]. Two weaker and broad structures located at about 719 and 732 eV were further detected, ascribable to the satellites peculiar to Fe^3+^ ions [95,96,98,99,100,101,102]. These findings fairly agreed with the results of the XRPD and MRS analyses.

The high-resolution photoelectron spectra of Ti^0^ 2*p* and Mn^0^ 2*p* core levels in nanocomposites α-Fe_2_O_3_(:D)@rGO-*w* are shown in Figure 3b,c, respectively. No contribution at 453.9 eV was detected in the Ti^0^ 2*p* photoelectron spectra of nanocomposites α-Fe_2_O_3_:Ti@rGO-*w* (Figure 3b), which allows ruling out the presence of elemental titanium (Ti^0^ [103,104]) in the hematite lattice. This finding, together with the BE positions of the 2*p*_1/2_ and 2*p*_3/2_ spin-orbit components reported in Table S2, strongly pointed at the spontaneous Ti ionization. Ti ions with +4 oxidation state [100,104,105,106,107] would be incorporated in the hematite lattice with electron transfer from the dopant to a surrounding Fe atom [90] (n-doped α-Fe_2_O_3_) and potentially improved electrical conductivity of hematite [108].

The 2*p*_1/2_ and 2*p*_3/2_ spin-orbit components of the Mn 2*p* core level in nanocomposite α-Fe_2_O_3_:Mn@rGO-30 were located at 653.4 and 641.8 eV, respectively (Figure 3c). Although these BE positions have been frequently reported for Mn ions with +2 valence state [109,110,111,112,113,114,115], the presence of ions with higher oxidation state (+3 or even higher) cannot be ruled out at all [112,113,116]. In general, the value of the spin-orbit spitting between the Mn 2p_3/2_ and Mn 2p_1/2_ components ranges from about 11.0–11.2 eV for Mn (0) to 11.8–11.9 eV for Mn (IV), while a BE of 640.3–644.5 eV for the Mn 2p_3/2_ core level indicates the presence of manganese ions with oxidation state varying from Mn^2+^ to Mn^4+^ [112,113,116]. Therefore, in sample α-Fe_2_O_3_:Mn@rGO-30, the BE of Mn 2p_3/2_ peak (641.8 eV) and the spin-orbit spitting (11.6 eV) prefigure an average oxidation number of about +3.3 (Table S3). Accordingly, the results of deconvolution of the Mn 2p_3/2_ peak (Figure S4) using Gaussian–Lorentzian model functions centered at 640.4, 642.2 and 645.8 eV (associated to MnO, MnO_2_ oxides species and Mn^4+^ ions in interaction with surrounding Fe ions, respectively) reflect an atomic abundance of Mn^2+^ ions of circa 40%. No Mn^0^ (contributing at a BE of 638.6 eV [103]) was detected, confirming the spontaneous ionization of the dopant.

Table S4 reports the surface elemental composition of the samples, as resulting from the quantitative analysis of the high-resolution photoelectron spectra. The O_C_/C atomic ratio of their carbonaceous component, estimated by assuming that hematite is the only iron-based crystalline phase that is formed, is reported in Table 1. In the case of nanocomposites based on Ti-doped hematite, its value did not significantly differ from that obtained for the α-Fe_2_O_3_@rGO-*w* ones (0.3), whereas the O_C_/C atomic ratio of α-Fe_2_O_3_:Mn@rGO-30 it was remarkably lower (<0.1), hinting at a higher reduction degree of GO.

The relative amount of the undoped/doped hematite in the nanocomposites (Table 1), as estimated via the quantitative analysis of the photoelectron spectra, always exceeds the α-Fe_2_O_3_(:D) content inferred from TGA. This finding is consistent with the picture of the nanomaterials provided by SEM analysis, with the α-Fe_2_O_3_(:D) nanoparticles homogeneously decorating the surface of graphene sheets.

### 3.2. Electrochemical Behavior

Previous studies had demonstrated the positive effect of the rGO component on the rate capability of the electrode and the crucial importance of the intimate contact between hematite NPs and rGO sheets [76], with the rate capability improving in the order bare α-Fe_2_O_3_ < physical mixture α-Fe_2_O_3_+rGO with 50 wt % rGO < nanocomposite α-Fe_2_O_3_@rGO-50. Moreover, it had been previously shown that doping of electrospun iron oxide nanofibers with an aliovalent impurity (silicon) causes the specific capacity to undergo a nearly fourfold increase at any rate between C/20 and 2 C [64].

The potential/capacity profiles of the phases were little influenced by the dopants or the amount of rGO and had the typical conversion/pseudocapacitive behavior already observed for these materials (Figure S5) [64,75]. All the doped compounds show a large irreversibility (40–50% of the total capacity) between the first sodiation and desodiation due to both the conversion reaction and the formation of the solid electrolyte interphase (SEI) layer. After the first cycle, the profiles stabilized and, during the sodiation, show a sloping zone (from 2.5 to 0.25 V vs. Na^+^/Na), which is typical of the pseudocapacitive behavior, followed by a more flat region (<0.25 V), which is the fingerprint of the conversion reaction.

**Figure 4:**
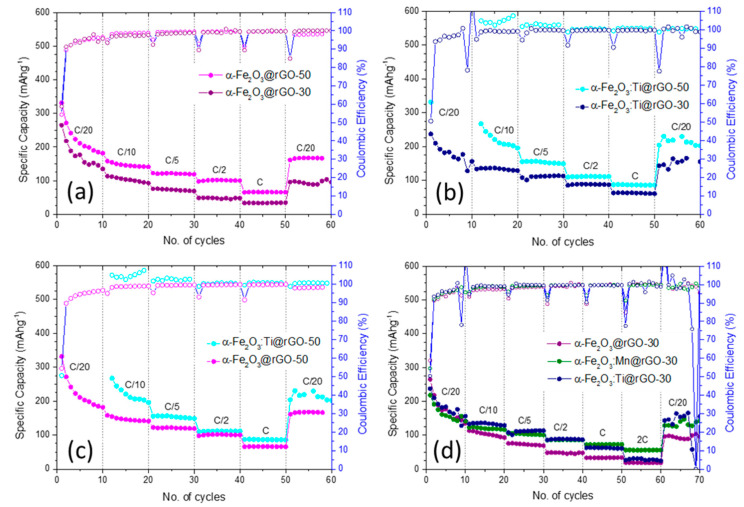
Effect of (**a**,**b**) the rGO amount and (**c**,**d**) the doping on the rate capabilities of all the nanocomposites.

Figure 4 compares the rate capabilities of all the nanocomposites pointing out the effect of the rGO amount (Figure 4a,b) and the doping (Figure 4c,d). The test consists in 60 cycles at different current rates, starting from the lowest current (C/20), then increasing the current four times each 10 cycles (C/10, C/5, C/2 and 1C) and finishing with C/20 again to prove the electrode integrity after fast cycling. The C rate have been calculated considering the total reduction of Fe(III) to metallic Fe, i.e., 1C = 503 mAg^−1^, a value almost double compared to the best carbonaceous materials (about 250 mAg^−1^). Specific capacities are related to the total amount of active material (80% of electrode film), because it is not possible to separate the capacitive contribution of graphene from that of the oxide. Moreover, with the oxide ratio being between 53 and 66% in the electrodes (see Table 1), the specific capacity normalization on the oxide amount alone would lead to higher but misleading and unpractical values.

Liu et al. [74] utilized a microwave-assisted method to synthesize Fe_2_O_3_@rGO composites with rGO contents in the range 10–50 wt %. The evaluation of the electrochemical behavior of these composites as anode materials for SIBs revealed a non-monotonic dependence of their specific capacity on the rGO content, with the best performance obtained for the composite α-Fe_2_O_3_@rGO-30 regardless of the rate (0.1–2 Ag^−1^) [74]. In the present case (Figure 4a), however, the specific capacity of α-Fe_2_O_3_@rGO-50 always exceeded that of α-Fe_2_O_3_@rGO-30, whose performance did not significantly differ from that of the bare oxide [76], particularly at higher rates. The same trend was confirmed for the material doped with Ti, however the effect was less important at high current (Figure 4b). It is clear that the addition of rGO to the oxide had two opposite effects: from one side, it increased the electronic transport properties by better connecting electrode particles; from the other it reduced the amount of the high capacity component of the electrode. In this case, being the specific capacity lower than the theoretical one, it seems more important the former aspect compared to the latter one.

Figure 4c compares the rate capabilities of nanocomposites α-Fe_2_O_3_@rGO-50 and α-Fe_2_O_3_:Ti@rGO-50. Although the improvement presently obtained was decidedly more restrained with respect to the case of electrospun Fe_2_O_3_:Si nanofibers, it was clearly evident that the introduction of a dopant in the hematite lattice bettered the electrochemical performance of the nanocomposite α-Fe_2_O_3_@rGO-50, as it translated into an increased specific capacity at rates below C. The Coulombic efficiency (CE) slightly improved too. The effect can be better understood considering the impedance of the two materials, which was measured by EIS in a proper three electrode cell (see SM Figure S6 and discussion thereof). At OCV, both the oxide/electrolyte interfaces had similar EIS spectra related to a pseudo-Randles circuit made by a serial resistance (the high frequency intercept) in series with a parallel RC circuit including a Z diffusive element. The serial resistance was associated with the electrolyte conductivity and, as expected, was similar for both the cells. In the parallel circuit, the resistance was related to the charge transfer resistance (RCT) of the electrochemical process, C was the double layer capacitance while Z the diffusion of the ions through the interface. The qualitative analysis of the parallel elements revealed that the most important difference between the two spectra was the value of the RCT, which was much lower for the Ti-doped iron oxide compared to the pristine one (112 vs. 183 Ω). The calculated exchange current densities, considering a mono-electronic process, were 1.8 × 10^-4^ A cm^−2^ and 2.9 × 10^−4^ A cm^−2^ for the α-Fe_2_O_3_@rGO-50 and α-Fe_2_O_3_:Ti@rGO-50, respectively. A larger exchange current density in oxide-based electrodes was usually associated to a better electronic conductivity of the material, in agreement with expectations [108].

In the case of the electrospun nanofibers, Si-doping provoked deep changes in the iron oxide structural, morphological and crystallographic properties: it suppressed large crystalline domains, led to the formation of disordered/amorphous nanosized elongated structures and promoted the formation of maghemite phase (γ-Fe_2_O_3_) in place of α-Fe_2_O_3_. These modifications mitigate the pulverization effect in the conversion reaction of the material, without lowering the electron conductivity of the doped material. No similar change was observed in the present case, which might be the reason for the more limited improvement observed.

The positive effect of the Ti doping was also confirmed at a lower rGO amount (30%), indeed the electrochemical behavior of the Ti-doped composite α-Fe_2_O_3_:Ti@rGO-30 was positively compared with that of the undoped one α-Fe_2_O_3_@rGO-30 (Figure 4d). At low rates (C/20–C/5), the introduction of Ti^4+^ impurity in the hematite lattice improves both the electrode stability and the CE. Analogously, Mn-doping of the hematite led to an improvement with respect to the pristine composite (Figure 4d). Moreover, after 60 cycles at rate increasing from C/20 to 2C, the anodes based on α-Fe_2_O_3_:Mn@rGO-30 and α-Fe_2_O_3_:Ti@rGO-30 respectively recovered about 90% and 83% of their average capacity at the lowest rate, exhibiting a remarkably increased stability with respect to the anode based on the composite α-Fe_2_O_3_@rGO-30, for which the recovery was only 54%.

Interestingly, the anode based on α-Fe_2_O_3_:Mn@rGO-30 exhibited remarkably higher stability at higher rates. At a rate of 2 C, it still retained a specific capacity (56 mAhg^−1^) higher by a factor of 2 and 3 compared to those of α-Fe_2_O_3_:Ti@rGO-30 and α-Fe_2_O_3_@rGO-30 at the same rate. Different from titanium that was incorporated in the hematite lattice as ions with a +4 oxidation state, with an electron transfer to surrounding Fe atoms (n-doping), manganese that is multivalent in nature can exist as Mn^2+^ and Mn^4+^ ions and it is a potential p-type or n-type dopant for α-Fe_2_O_3_ [117]. Its incorporation in the hematite lattice may change the distance between some crystallographic planes [118] and, besides, it is beneficial for the electrical conductivity [87,117]. The occurrence of similar changes might be responsible for the improved stability of the nanocomposite α-Fe_2_O_3_:Mn@rGO-30 with respect to the pristine α-Fe_2_O_3_@rGO-30.

## 4. Conclusions

Physicochemical characterization and electrochemical testing of α-Fe_2_O_3_@rGO, α-Fe_2_O_3_:Ti@rGO and α-Fe_2_O_3_:Mn@rGO nanocomposites, prepared by the one-step solvothermal method, evidenced that:

- Titanium was incorporated in the hematite lattice as Ti^4+^ ions with electron transfer to surrounding Fe atoms (n-doping), whereas the type (p- or n-) of doping by manganese could not clearly be assessed due to is multivalent nature;
- The doping did not influence the crystalline phase and morphology of the iron oxide nanoparticles anchored on the rGO sheets;
- Conversely, it remarkably improved the electrochemical performance with respect to the anode based on the composite α-Fe_2_O_3_@rGO;
- For fixed rGO content, the α-Fe_2_O_3_:Ti@rGO-based anodes exhibited better rate capability at lower rates, whereas α-Fe_2_O_3_:Mn@rGO-based anodes show enhanced stability at higher rates, still retaining 56 mAhg^−1^ at a rate of 2 C;
- Increasing the rGO content of the nanocomposites from 30 to 50 wt % was beneficial to a specific capacity at any rate.

## Figures and Tables

**Table 1:** Results of TGA, XPS and XRD analyses.

Samples Code	rGO Content (wt %)		α-Fe_2_O_3_(:D) Content (wt %)		O_C_/C	d (nm) ^a^
	Nominal	Measured (TGA)	Measured (TGA)	Measured (XPS)	XPS	XRPD
α-Fe_2_O_3_@rGO-50	50	46.7	53.3	59.4	0.319	27.2
α-Fe_2_O_3_:Ti@rGO-50	50	48.3	51.7	55.8	0.323	17.3
α-Fe_2_O_3_@rGO-30	30	33.5	66.5			28.7
α-Fe_2_O_3_:Ti@rGO-30	30	41.2	58.8	68.7	0.285	17.8
α-Fe_2_O_3_:Mn@rGO-30	30	38.9	61.1	66.5	0.091	29.1

The rGO and α-Fe_2_O_3_ contents measured via thermogravimetric analysis (TGA) are compared with the nominal ones and the measured via X-ray photoelectron spectroscopy (XPS) ones, respectively. The surface oxidation degree of rGO, as monitored by the carbon-bonded oxygen (O_C_) to the carbon atomic ratio, estimated via XPS, is also reported, as well as the average size of the hematite crystallites, as inferred via the Scherrer equation from the X-ray powder diffraction (XRPD) patterns. ^a^ d = diameter

## Supplementary Materials

The following are available online at https://www.mdpi.com/2079-4991/10/8/1588/s1, Figure S1: Morphology changes produced by the reduction of (a) GO to (b) rGO upon solvothermal treatment, as displayed by the SEM analysis. Figure S2: Obtainment of GO and its subsequent reduction to rGO, as ascertained by means of (a) MRS, (b) XRPD and (c) XPS. Figure S3: Results of TGA. Table S1: Results of TGA. Table S2: Binding energies and energy splitting of the spin-orbit components of Fe 2p, Ti 2p and Mn 2p core levels. Figure S4: Results of deconvolution of Mn 2p_3/2_ core level in the composite α-Fe_2_O_3_:Mn@rGO30. Table S3: Results of deconvolution of Mn 2p core level in the composite α-Fe_2_O_3_:Mn@rGO30. Table S4: Surface elemental composition of the samples (from XPS spectra). Figure S5: Potential/capacity profiles of electrodes based on composites (a) α-Fe_2_O_3_@rGO-30, (b) α-Fe_2_O_3_:Ti@rGO-30 and (c) α-Fe_2_O_3_:Mn@rGO-30. Figure S6: Nyquist plots for electrodes based on composites α-Fe_2_O_3_@rGO-50 and α-Fe_2_O_3_:Ti@rGO-50.
