# Supplementary material for: Effect of Hematite Doping with Aliovalent Impurities on the Electrochemical Performance of α-Fe2O3@rGO-Based Anodes in Sodium-Ion Batteries
Source: Nanomaterials (Basel). 2020 Aug 12;10(8):1588. doi: 10.3390/nano10081588 (PMC7466594; doi:10.3390/nano10081588)
Supplement: Supplementary file 1 [file nanomaterials-10-01588-s001.pdf]

nanomaterials

Supplementary materials

Effect of Hematite Doping with Aliovalent Impurities on the Electrochemical Performance of α-Fe_2O_3@rGO-Based Anodes in Sodium-Ion Batteries

Vincenza Modafferi [1], Claudia Triolo [1], Michele Fiore [2], Alessandra Palella [3], Lorenzo Spadaro [3], Nicolò Pianta [2], Riccardo Ruffo [2], Salvatore Patanè [4], Saveria Santangelo [1,5,*] and Maria Grazia Musolino [1,*]

1 Dipartimento di Ingegneria Civile, dell'Energia, dell'Ambiente e dei Materiali (DICEAM), Università Mediterranea di Reggio Calabria, 89122 Reggio Calabria, Italy; vincenza.modafferi@unirc.it (V.M.); claudia.triolo@unirc.it (C.T.)

2 Dipartimento di Scienza dei Materiali, Università di Milano Bicocca, 20125 Milano, Italy; m.fiore11@campus.unimib.it (M.F.); n.pianta@campus.unimib.it (N.P.); riccardo.ruffo@unimib.it (R.F.)

3 Istituto di Tecnologie Avanzate per l'Energia (ITAE) del Consiglio Nazionale delle Ricerche (CNR), 98126 Messina, Italy; alessandra.palella@itae.cnr.it (A.P.); lorenzo.spadaro@itae.cnr.it (L.S.)

4 Dipartimento di Scienze Matematiche e Informatiche, Scienze Fisiche e Scienze della Terra (MIFT), Università di Messina, 98166 Messina, Italy; patanes@unime.it

5 Consorzio Interuniversitario Nazionale per la Scienza e Tecnologia dei Materiali (INSTM), 50121 Firenze, Italy

* Correspondence: saveria.santangelo@unirc.it (S.S.); mariagrazia.musolino@unirc.it (M.G.M.); Tel.: +39-0965-1692305 (S.S.); +39-0965-1692312 (M.G.M.)

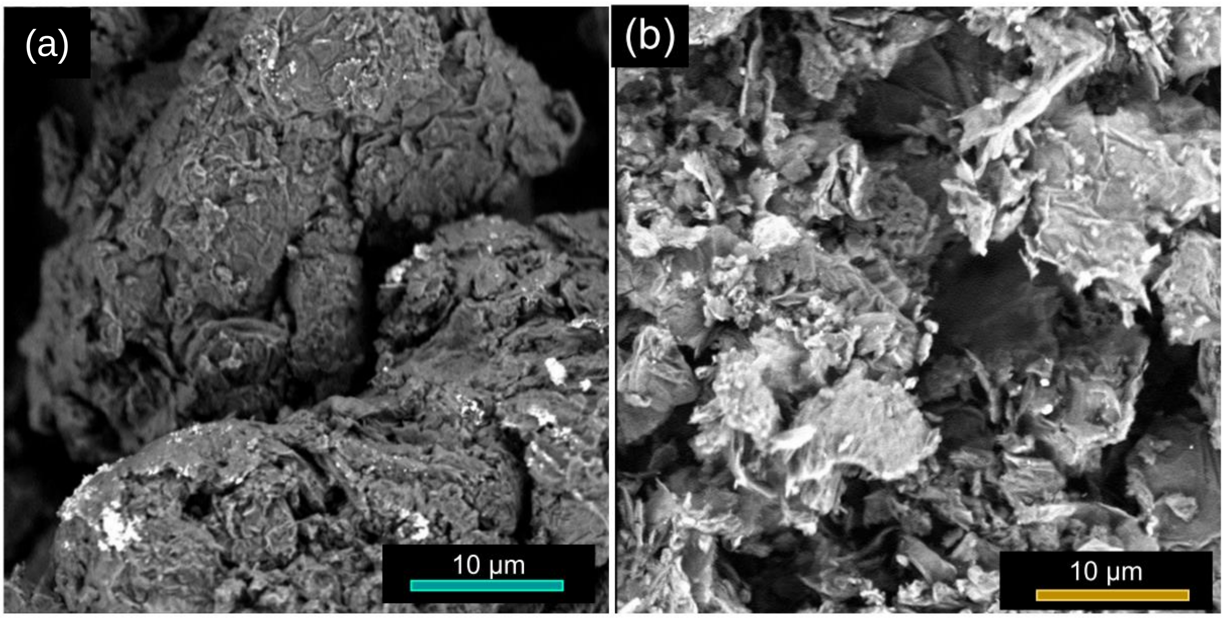

Figure S1. Morphology changes produced by the reduction of (**a**) GO to (**b**) rGO upon solvothermal treatment, as displayed by SEM analysis.

Reduction of GO to rGO during the solvothermal treatment

After the solvothermal treatment, i) the G-band shifted (from 1600 to 1588 cm^{-1}) and the D/G intensity ratio increased (from 2.75 to 3.01), indicating a diminishing of the oxidation degree and a partial restoration of the π network (Figure S2a); ii) the very broad (002) band at 2θ = 24.1°, typical of disordered graphitic carbons, replaced the narrow (001) peak at 2θ = 9.5° peculiar to GO (Figure S2b), proving the *d*-spacing reduction (from 0.955 to 0.369 nm); and iii) the profile of the C 1*s* core level dramatically changed (Figure S2c), confirming the removal of oxygenated functionalities, with O/C atomic ratio decrease (from 0.49 to 0.32). For further details see ref. [76]. Also the morphology underwent visible changes, as proven by the SEM images (Figure S1).

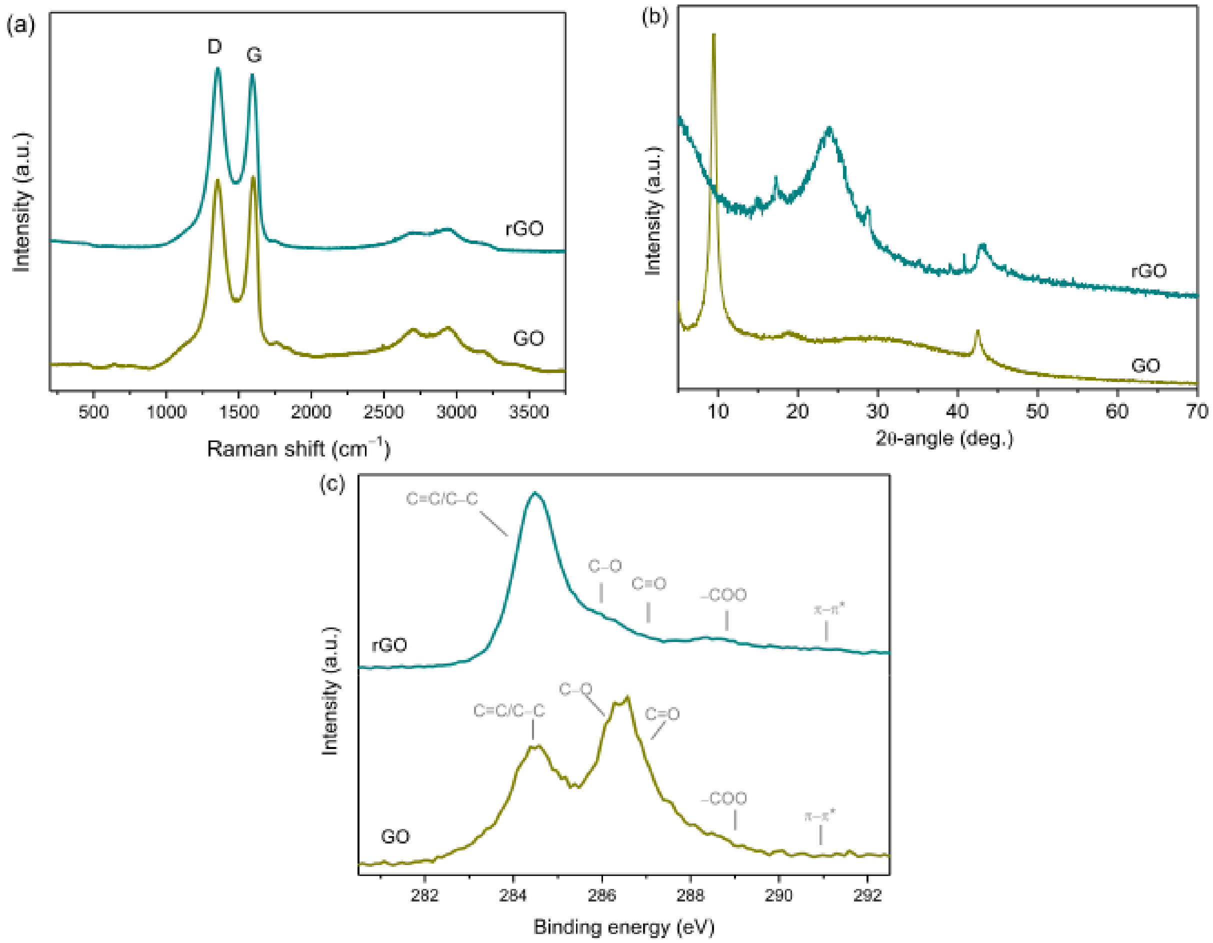

Figure S2. Obtainment of GO and its subsequent reduction to rGO, as ascertained by means of (**a**) MRS, (**b**) XRPD and (**c**) XPS.

Measured rGO content of the nanocomposites

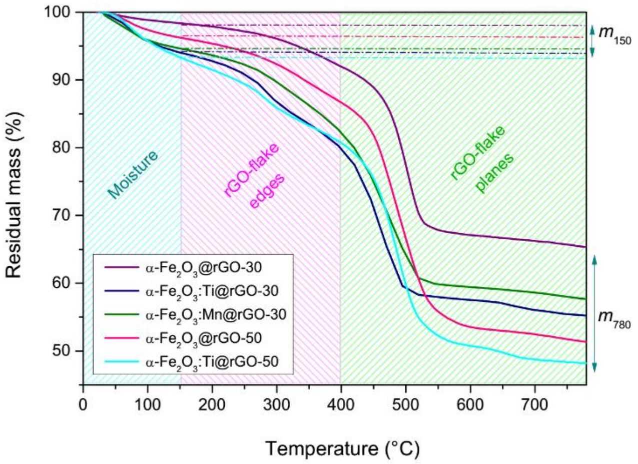

Figure S3. Results of TGA. Three main temperature ranges are singled out, corresponding to the release of adsorbed water ($T \leq 150$ °C) and the complete combustion of the rGO flakes, starting from their edges (150 °C < $T \leq 400$ °C) and subsequently extending to the graphenic planes (400°C < $T \leq 780$ °C). For $T > 780$ °C, the residual mass does not change any more.

Table S1. Results of TGA. m_{150} and m_{780} denote the residual masses at 150 and 780 °C, i.e. after the loss of the adsorbed water and the complete combustion of rGO, respectively. The residual mass, m_{150}–m_{780}, after the moisture release is considered and the rGO and α-Fe_2O_3(:D) contents of the nanocomposites are estimated as $\frac{m_{150}-m_{780}}{m_{150}} \cdot 100$ and $\frac{m_{780}}{m_{150}} \cdot 100$, respectively.

Samples code	Nominal rGO content (wt%)	m_{150} (wt%)	m_{780} (wt%)	m_{150}–m_{780} (wt%)	Measured rGO content (wt%)	Measured α-Fe_2O_3 content (wt%)
α-Fe_2O_3@rGO-50	50	96.2	51.3	44.9	46.7	53.3
α-Fe_2O_3:Ti@rGO-50	50	93.3	48.2	45.1	48.3	51.7
α-Fe_2O_3@rGO-30	30	98.4	65.4	33.0	33.5	66.5
α-Fe_2O_3:Ti@rGO-30	30	94.1	55.3	38.8	41.2	58.8
α-Fe_2O_3:Mn@rGO-30	30	94.7	57.8	36.9	38.9	61.1

Surface composition and chemical environment of the component species in the nanocomposites

Table S2. Binding energies (in eV) and energy splitting (ΔE) of the two spin-orbit components of Fe 2*p*, Ti 2*p* and Mn 2*p* core levels in the nanocomposites.

Sample code	Fe 2*p*			Ti 2*p*			Mn 2*p*		
	$2p_{1/2}$	$2p_{3/2}$	ΔE	$2p_{1/2}$	$2p_{3/2}$	ΔE	$2p_{1/2}$	$2p_{3/2}$	ΔE
α-Fe_2O_3@rGO-50	724.8	711.3	13.6						
α-Fe_2O_3:Ti@rGO-50	725.5	711.6	13.9	464.6	458.9	5.7			
α-Fe_2O_3:Ti@rGO-30	725.2	711.5	13.6	464.7	458.8	5.9			
α-Fe_2O_3:Mn@rGO30	724.9	711.3	13.6				653.4	641.8	11.6

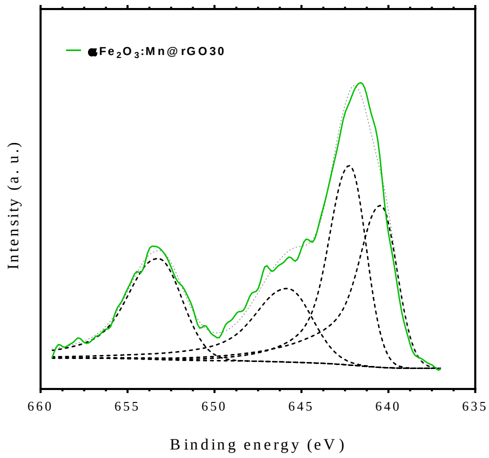

Figure S4. Results of deconvolution of Mn $2p_{1/2}$ core–level in the composite α-Fe_2O_3:Mn@rGO-30.

Table S3. Results of deconvolution of Mn 2p core–level aimed at estimating the average oxidation number (AON) of manganese in the composite α-Fe_2O_3:Mn@rGO-30.

Sample code	Mn $2p_{3/2}$		Mn $2p_{1/2}$		ΔeV	Mn AON	Peak 1		Peak 2		Peak 3	
							Mn^{2+}		Mn^{4+}		Mn^{4+}*	
	eV	A	eV	A			eV	%A	eV	%A	eV	%A
α-Fe_2O_3:Mn@rGO-30	653.4	3326	641.8	945	11.6	3.3	640.43	40.05%	642.21	39.06%	645.79	20.90%

* Mn^{4+} ions in interaction with surrounding Fe ions.

Table S4. Surface elemental composition of the samples, as inferred via the quantitative analysis of the x-ray photoelectron spectra. O_T stands for the surface oxygen present in the nanocomposite, while D denotes the dopant. O_C indicates the carbon-bonded oxygen, estimated by assuming that hematite is the only iron-based crystalline phase formed.

Sample code	Atomic concentration (at%)				Concentration (wt%)				
	C	O_T	Fe	D	C	O_T	O_C	Fe	D
α-Fe_2O_3@rGO-50	47.6	37.5	14.9	0.0	28.5	30.0	12.1	41.5	0.0
α-Fe_2O_3:Ti@rGO-50	50.4	35.2	12.6	1.8	30.9	28.8	13.3	35.9	4.4
α-Fe_2O_3:Ti@rGO-30	41.9	37.9	17.3	3.0	22.7	27.3	8.6	43.5	6.5
α-Fe_2O_3:Mn@rGO30	52.0	30.4	17.1	0.5	29.9	23.2	3.6	45.6	1.3

Electrochemical behaviour of the nanocomposites

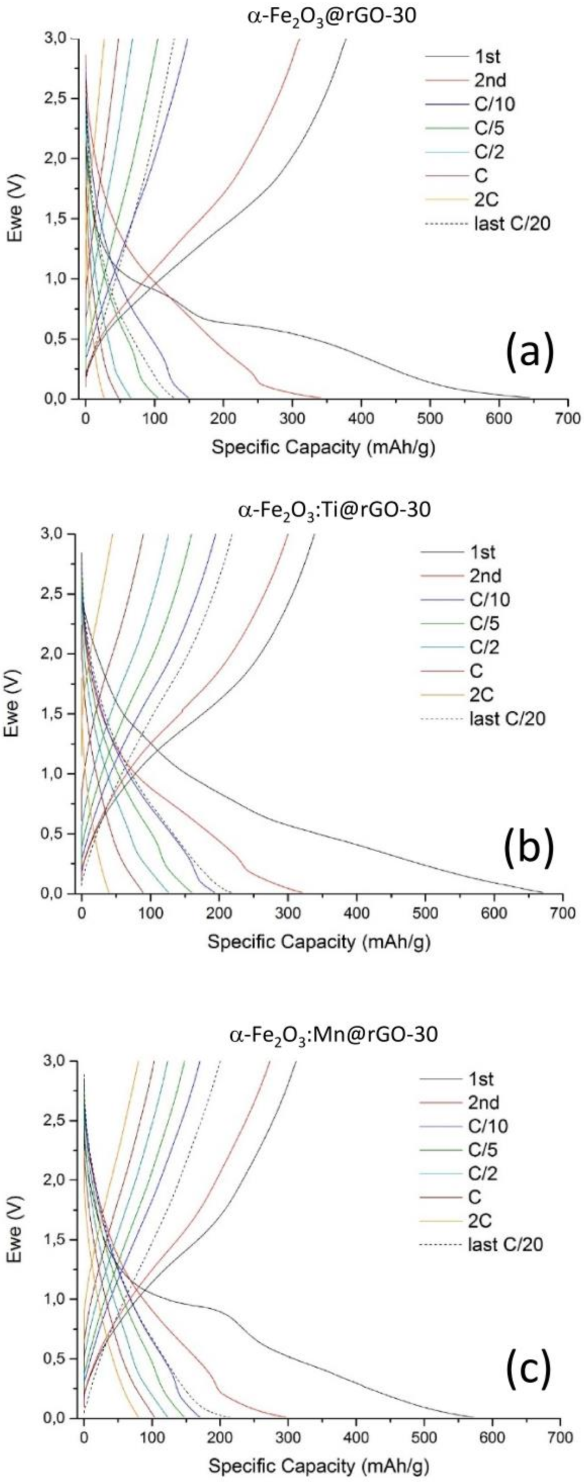

Figure S5. Potential/capacity profiles of electrodes based on composites (**a**) α-Fe_2O_3@rGO-30, (**b**) α-Fe_2O_3:Ti@rGO-30 and (**c**) α-Fe_2O_3:Mn@rGO-30.

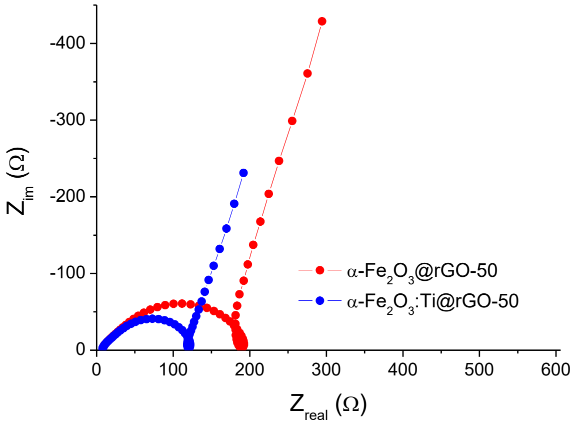

Figure S6. Nyquist plot of three-electrode cell equipped with *α*-Fe_2O_3@rGO-50 and *α*-Fe_2O_3:Ti@rGO-50.
